# Association between the intake of potentially risky beverages and the occurrence of endometrial polyps: a case–control study

**DOI:** 10.3389/fnut.2025.1538405

**Published:** 2025-02-04

**Authors:** Rui Fu, Shipeng Zhang, Chang Cai, Xiaocui Wang, Yanjie Jiang, Xiulian Zhuang, Jiating Zhang, Xiaoli Ji, Chengcheng Yang

**Affiliations:** ^1^Clinical Medical College, Chengdu University of Traditional Chinese Medicine, Chengdu, Sichuan, China; ^2^Hospital of Chengdu University of Traditional Chinese Medicine, Chengdu, Sichuan, China; ^3^Nanjing Hospital of Chinese Medicine Affiliated to Nanjing University of Chinese Medicine, Nanjing, Jiangsu, China; ^4^Liaoning University of Traditional Chinese Medicine Xinglin College, Shenyang, Liaoning, China

**Keywords:** endometrial polyps, potentially risky beverages, anxiety, female, logistic

## Abstract

**Background:**

This case–control study aimed to examine the association between the frequency of potentially risky beverage consumption, levels of anxiety, and the prevalence of endometrial polyps.

**Methods:**

A total of 418 participants were enrolled in the study, comprising 206 cases and 212 controls. The case group consisted of patients who visited the gynecological clinic at the Affiliated Hospital of Chengdu University of Traditional Chinese Medicine and were diagnosed with endometrial polyps (Eps) based on international diagnostic criteria. The control group consisted of women of childbearing age who visited the gynecological clinic with similar clinical symptoms but did not have EPs. Basic information, consumption of potentially risky beverages (PRB), and anxiety levels for both groups were collected through a questionnaire survey. Finally, the relationship between the frequency of PRB consumption, anxiety levels, and the prevalence of EPs was evaluated.

**Results:**

In this study, we identified a significant positive association between the consumption of PRB and the prevalence of EPs. PRB intake was categorized into three groups based on the cumulative total score: 5–8 for the Low potentially risky beverages (LPRB) intake group, 9–12 for the medium potentially risky beverages (MPRB) intake group, and 13–21 for the high potentially risky beverages (HPRB) intake group. The results revealed that PRB consumption frequency was significantly associated with EPs (OR: 2.348, 95% CI: 1.153–4.78), with higher PRB intake correlating with an increased risk of EPs (*p*-value: 0.014). However, no significant difference was observed between the LPRB, MPRB, HPRB intake frequency groups and the different levels of anxiety (*p*-value: 0.793).

**Conclusion:**

Increased consumption of PRB was clearly associated with a greater risk of EPs, and over half of the participants exhibited varying degrees of anxiety. These findings suggest that the risk of EPs can be mitigated by controlling beverage intake and highlight the need for increased attention to women’s mental health.

**Clinical trial registration:**

NCT06295510.

## Introduction

Endometrial polyps (EPs) are common gynecological conditions and represent a frequent intrauterine lesion resulting from local hyperplasia of the endometrium, primarily affecting women of childbearing age. Epidemiological studies indicate that EPs account for 24 to 25% of gynecological diseases in Chinese women (1). Moreover, the recurrence rate of Eps is high (2). The incidence of EPs is age-dependent, contributing to 10–40% of abnormal uterine bleeding before menopause and 10.1–38.0% after menopause (3). Most scholars believe that the pathogenesis of EPs is related to hormonal imbalances and inflammation and that factors such as obesity, advanced age, estrogen stimulation, and angiogenesis are closely related to the occurrence and progression of endometriosis (4). The clinical symptoms of EPs are not typical and include heavy menstrual bleeding, intermenstrual bleeding, and prolonged menstrual duration. EPs not only affect the quality of life of patients but are also are directly associated with reduced fertility (5). Studies have shown that EPs can obstruct fallopian tube opening and occupy space in the uterine cavity, thus obstructing sperm transport and embryo implantation. In addition, the local inflammatory response mediated by EPs affects sperm survival and inhibits embryo implantation and development. Patients with EPs experience reduced endometrial receptivity and sexual intercourse frequency, ultimately resulting in lower pregnancy rates (6).

According to the most recent survey, the probability of a combined diagnosis of EPs in women with unexplained infertility ranges from 16.5 to 26.5%, while the incidence of primary infertility ranges from 3.8 to 38.5%, and the incidence of secondary infertility ranges from 1.8 to 17% (7). Currently, there is no effective treatment for EPs. Hysteroscopic endometrial resection is the preferred method for treating EPs, but postoperative recurrence rates remain high, ranging from 33.33 to 52% (8); as follow-up time increases, the recurrence rate of EPs also rises. Consequently, identifying effective prevention and treatment strategies has become an urgent issue that requires resolution.

A large number of studies have shown that changes in dietary structure and intake are important factors in disease prevention. Although no direct link has been found between dietary intake and EPs, several studies indicate a significant association between different diets and endometrial-related diseases. Sakine Ghasemisedaghat et al. reported that the consumption of plant protein and vitamin K could reduce the incidence of endometriosis, whereas the consumption of animal protein, haem iron, and a high glycemic load was associated with an increased incidence of endometriosis (9). SHIVAPPA N et al. reported a moderate positive correlation between high glycemic load (but not the glycemic index) and endometrial cancer (10).

In recent years, more attention has been paid to the adjustment of dietary structure, but the harm of beverage intake to health has been ignored. The increase of beverage intake is also an important risk factor leading to the increase of chronic diseases. Studies have shown that milk tea, carbonated drinks, and sugar-sweetened beverages are significantly associated with increased depression in college students (11), carbonated drinks have been linked to osteoporosis and type 2 diabetes (12); sugar-sweetened beverages have shown the strongest harmful effects, with clear associations with various health outcomes (13); caffeine intake is associated with fluctuations in estrogen levels (14); and soy milk, which contains a high concentration of isoflavones, is related to hormonal fluctuations, including estrogen levels, in women (15). The high contents of sugars, unsaturated fats, additives, hormones, proinflammatory substances, and other components in these beverages may pose significant health risks (16). Considering the varying degrees of harm these five beverages may cause to human health, carbonated drinks, soy milk, sugar-sweetened beverages, milk tea, and coffee were classified as PRB in this study (12). This study evaluated the intake of these beverages and examined the correlation between their consumption and the incidence of EPs. There is a well-established relationship between the occurrence of EPs and the levels of hormones as well as proinflammatory substances (17). It can be speculated that the increased intake of these PRB may be a key factor contributing to the rising incidence of EPs. This study aims to clarify the relationship between the incidence of EPs, the intake of PRB, and anxiety levels, thereby providing evidence-based insights to assist in dietary recommendations and the prevention of EPs among female patients.

## Methods

### Study design, population, and sample size

In this study, women of childbearing age who attended the gynecological outpatient department of the Affiliated Hospital of Chengdu University of Traditional Chinese Medicine between 1^st^ Mar 2024 and 1^st^ May 2024 were selected as study participants, and the sample size was determined via the following Equation 1,

(1) $$\mathrm{Sample}\;\mathrm{size}=\frac{r+1}{r}\;\frac{{\left(Z\beta +Za/2\right)}^{2}P\left(1-P\right)}{{\left(P1-P2\right)}^{2}}$$

**Figure 1:**
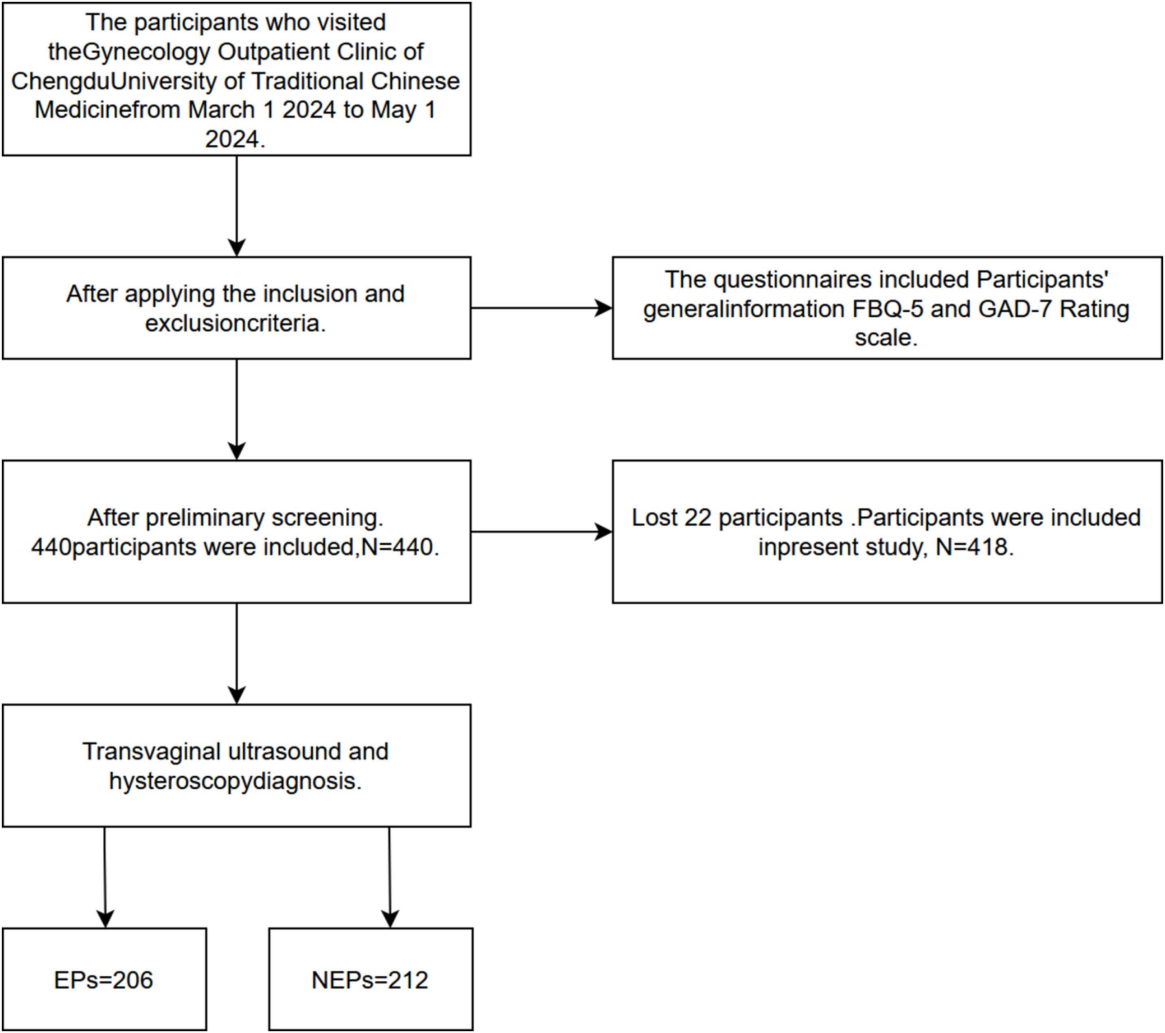
Experimental flow chart.

**Figure 2:**
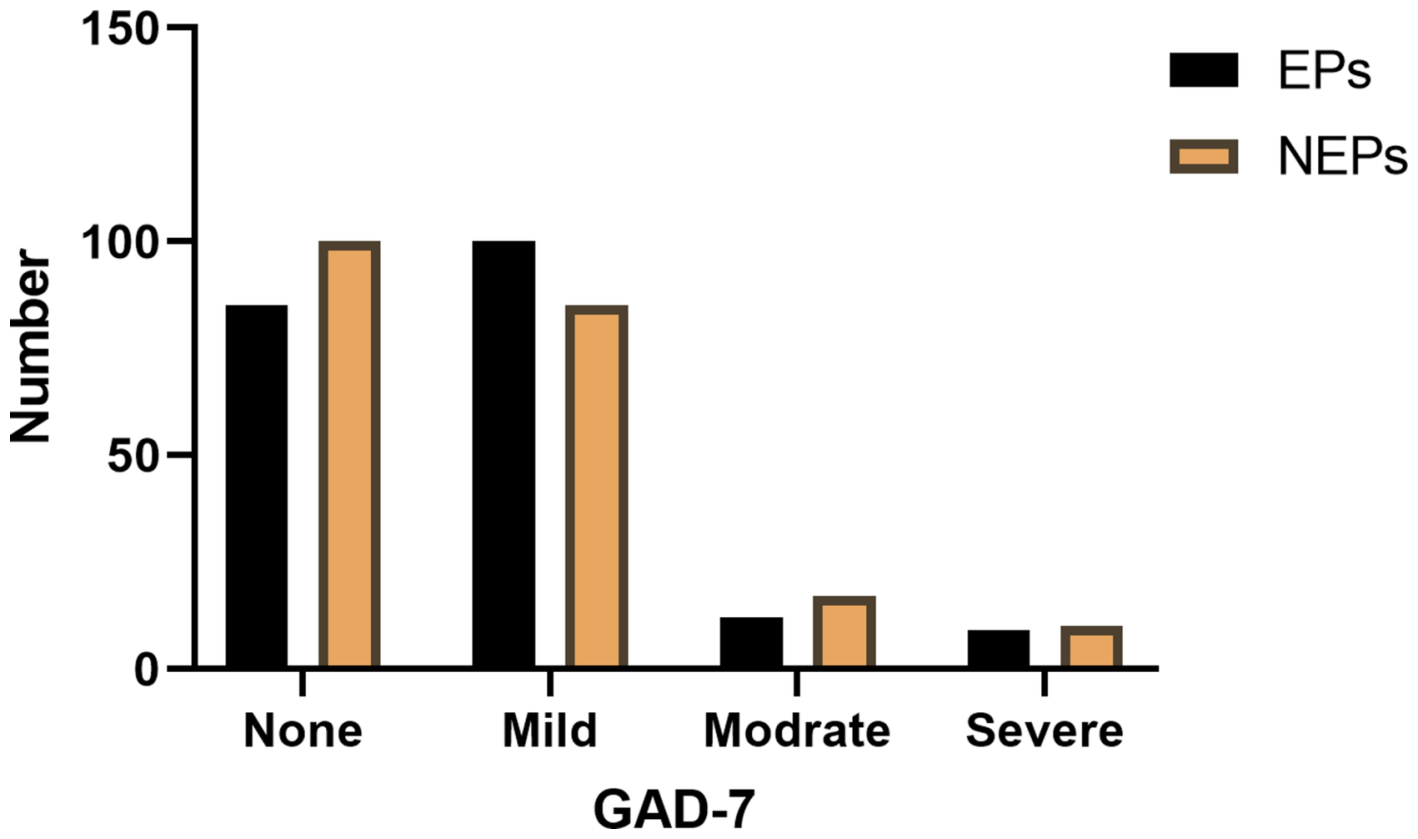
Two groups of anxiety frequency statistics.

In this study, r represents the case-to-control ratio, which is set to 1, indicating an equal number of cases and controls. Zα/2 represents the standard normal variable for a significance level of 0.05/2, which equals 1.96, whereas Zβ indicates a power of 90%, corresponding to a value of 1.28. P1 denotes the proportion of cases, specifically the prevalence of EPs reported in previous studies, which is 0.14. P2 represents the proportion of the control group. Following extensive discussions between the author and statisticians, it is assumed that the proportion of gynecological patients of childbearing age without EPs is 0.27. P is the average proportion of cases to the control group, which is calculated as P1 + P2. After calculation, the required sample size was determined to be 206. To ensure adequate age representation in matching cases and controls, as well as the validity of the sample size, we plan to recruit 220 cases and 220 controls. To mitigate potential recall bias, a contradictory response option was added to assess the accuracy of the questionnaire responses. If any discrepancies were identified between the presurvey and postsurvey answers, the corresponding questionnaire was excluded. Additionally, some participants expressed concerns about information security; as a result, 22 participants opted to withdraw from the survey. Consequently, the final sample consisted of 206 valid questionnaires from the case group and 212 from the control group (Figure 1). The tool used in Figure 1,^1^ The tool used in Figure 2 is GraphPad Prism 8.

### Inclusion and exclusion criteria

Women aged 18–49 years who present at the gynecologic clinic will be eligible for inclusion in this study. The inclusion criteria for the study group were as follows: (1) aged between 18 and 49 years; (2) had a diagnosis of EPs confirmed by transvaginal ultrasound; (3) had primary complaints, including intermenstrual bleeding, irregular menstruation, abnormal uterine bleeding, dysmenorrhea, or excessive menstrual bleeding; and (4) provided informed consent after the study’s purpose was explained. The exclusion criteria were as follows: (1) inability to accurately recall beverage intake 1 month prior; (2) accurate recall of beverage intake 1 month prior but with logical inconsistencies; (3) The use of hormonal medications for more than 1 month, including contraceptives, estrogen, progestins, and other related drugs; (4) history of long-term hormonal drug use; and (5) presence of significant mental disorders. Patients with similar complaints who do not have EPs will be assigned to the control group.

### Questionnaire design

#### General questionnaire

The general questionnaire collected basic demographic and lifestyle information, including height, weight, age, residence (with consideration of environmental pollution levels), education level, family income per capita, smoking history, alcohol consumption history, marital history, sexual history, and abortion history.

#### FBQ-5 beverage frequency scale

A self-designed Beverage Frequency Questionnaire (FBQ-5) was used to assess participants’ beverage consumption over the past month. To minimize recall bias, a contradictory option was included to enhance the accuracy of responses. The beverage categories included carbonated drinks, soy milk, sugar-sweetened beverages, milk tea, and coffee. Beverage consumption frequencies were classified as follows: ‘almost never,’ ‘occasionally’ (0–1 times per week), ‘sometimes’ (1–2 times per week), ‘often’ (3–4 times per week), and ‘always’ (5 or more times per week), with scores ranging from 1 to 5, where higher scores indicated more frequent consumption of PRB. The cumulative score ranged from 5 to 25. Based on quartile ranges, scores of 5–8 were categorized into the LPRB group, 9–12 into the MPRB group, and 13–25 into the HPRB group. The reliability and validity of the questionnaire were assessed, with a Kaiser-Meyer-Olkin (KMO) value of 0.893 and a Cronbach’s *α* coefficient of 0.788, indicating good construct validity.

#### GAD-7 anxiety rating scale

Psychological anxiety is prevalent among women, as indicated by several studies. Therefore, the Generalized Anxiety Disorder-7 (GAD-7) scale was used to assess the anxiety levels of participants in this study (18). This instrument has been validated as a reliable tool for assessing anxiety in the Chinese population, including pregnant women (19). Seven items with typical anxiety symptoms over the past 2 weeks were measured on a 4-point Likert-type scale: 0 = never, 1 = several days, 2 = more than half of the days, and 3 = nearly every day. A cut-off score of 5 or higher was considered to indicate anxiety (20). In this study, anxiety levels in women were assessed using seven questions from the GAD-7 scale. Based on the cumulative GAD-7 score, anxiety severity was classified as follows: none (0–4), mild (5–9), moderate (10–14), and severe (15–21).

### Statistical methods

Data analysis was conducted via SPSS 26.0 (IBM, Armonk, New York, United States). Cronbach’s alpha, Bartlett’s test of sphericity, and the Kaiser–Meyer–Olkin (KMO) test were employed to evaluate the reliability and validity of the FBQ-5 and GAD-7 scales. Continuous variables are expressed as the means ± standard deviations, and independent sample tests were used to compare differences between the means of continuous variables among cases and controls. Differences among multiple groups were assessed via analysis of variance (ANOVA). In cases of significant differences among groups, pairwise comparisons were conducted via the least significant difference (LSD) method. The Spearman correlation test was used to analyze the relationships among categorical variables. Multivariate logistic regression analysis was conducted to identify independent predictors of the prevalence of EPs. The significance level was set at *α* = 0.05 (two-tailed), and *p* < 0.05 was deemed statistically significant.

## Results

### General data statistics of enrolled patients

Table 1 presents the general characteristics of the case and control groups, as well as the frequency of consumption of five types of PRB. A total of 418 participants were included, with 206 in the EPs group and 212 in the nonendometrial polyps (NEPs) group. As shown in the table, the average age of the EPs group (29.63 ± 7.53) was significantly younger than that of the NEPs group (35.16 ± 8.94), suggesting that EPs tend to occur at a younger age. The incidence of EPs was also significantly associated with marital status (*p* < 0.001), environmental pollution (*p* < 0.001), education level (*p* = 0.015), sexual activity (*p* < 0.001), pregnancy history (*p* < 0.001), abortion history (*p* < 0.001), smoking history (*p* = 0.020), and reproductive history (*p* < 0.001), with all variables showing statistical significance (*p* < 0.05). However, no significant difference was observed between the different levels of anxiety and the low, medium, and high PRB intake groups (*p* = 0.793). Based on these findings, we conclude that, compared to the control group, patients with EPs tend to be younger, and women who are sexually inactive have a significantly lower probability of developing EPs compared to those who are sexually active. Additionally, women with higher education levels are less likely to have EPs than those with lower education levels. Moreover, the average cumulative scores for the frequency of PRB intake were significantly higher in the EPs group than in the control group (*p* = 0.038). However, no significant differences were found in the incidence of EPs with respect to family per capita monthly income (*p* = 0.268) or drinking history (*p* = 0.232).

**Table 1:** General characteristics and drinking frequency.

		Eps	NEPs	*p* value
Age (M ± SD)		29.63 ± 7.53	35.16 ± 8.94	<0.001**
Body Mass Index (M ± SD)		22.02 ± 3.56	22.16 ± 5.05	0.743
Marriage (N, %)	Married	48 (11.5%)	109 (26.1%)	<0.001**
	Single	158 (37.8%)	103 (24.9%)	
Environmental pollution (N, %)	Near the city	140 (33.5%)	89 (21.3%)	<0.001**
	Central city	66 (15.8%)	123 (29.4%)	
Degree of education	Junior high school and below	21 (5.0%)	7 (1.7%)	0.015*
	senior high school	18 (4.3%)	24 (5.7%)	
	University and above	167 (40.0%)	181 (43.3%)	
Per capita monthly household income	<3,000¥	58 (13.9%)	46 (11.0%)	0.268
	3,000–5,000¥	105 (25.1%)	113 (27.0%)	
	>5,000¥	43 (10.3%)	53 (12.7%)	
Smoking (N, %)	Yes	25 (12.1%)	12 (5.7%)	0.020 *
	No	181 (87.9%)	200 (94.3%)	
Drinking (N, %)	Yes	70 (34.0%)	84 (39.6%)	0.232
	No	136 (66.0%)	128 (60.4%)	
Sexual life (N, %)	Yes	190 (45.8%)	133 (31.8%)	<0.001**
	No	16 (3.8%)	79 (18.9%)	
Whether pregnant or not (N, %)	Yes	122 (29.2%)	73 (17.5%)	<0.001**
	No	84 (20.1%)	139 (33.3%)	
History of abortion (N, %)	Yes	90 (23.7%)	48 (11.5%)	<0.001**
	No	116 (27.8%)	164 (39.2%)	
Childbearing history (N, %)	Yes	99 (23.7%)	64 (15.3%)	<0.001**
	No	107 (25.6%)	148 (35.4%)	
Carbonate beverages (N, %)	almost never	22 (10.7%)	41 (19.3%)	0.088
	occasionally (0–1 times per week)	113 (54.9%)	106 (50%)	
	sometimes (1–2 times per week)	46 (22.3%)	39 (18.4%)	
	often (3–4 times per week)	20 (9.7%)	24 (11.3%)	
	always (4–5 times per week)	5 (2.4%)	2 (0.9%)	
Soybean milk (N, %)	almost never	56 (27.2%)	45 (21.2%)	0.165
	occasionally (0–1 times per week)	107 (51.9%)	132 (62.3%)	
	sometimes (1–2 times per week)	31 (15.0%)	20 (9.4%)	
	often (3–4 times per week)	10 (4.9%)	13 (6.1%)	
	always (4–5 times per week)	2 (1.0%)	2 (0.9%)	
Sugar-sweetened beverages (N, %)	almost never	29 (14, 1%)	54 (25.5%)	0.025*
	occasionally (0–1 times per week)	130 (63.1%)	125 (59.0%)	
	sometimes (1–2 times per week)	33 (16.0%)	26 (12.3%)	
	often (3–4 times per week)	10 (4.9%)	6 (2.8%)	
	always (4–5 times per week)	4 (1.9%)	1 (0.5%)	
Milk tea (N, %)	almost never	28 (13.6%)	54 (25.5%)	0.022 *
	occasionally (0–1 times per week)	138 (67.0%)	121 (57.1%)	
	sometimes (1–2 times per week)	35 (17.0%)	30 (14.2%)	
	often (3–4 times per week)	4 (1.9%)	7 (3.3%)	
	always (4–5 times per week)	1 (0.5%)	0 (0%)	
Coffee (N, %)	Never	86 (41.7%)	74 (34.9%)	0.091
	Occasionally (1–2 times a month)	73 (35.4)	94 (44.3%)	
	Frequently (1–2 times a week)	22 (10.7%)	30 (14.2%)	
	Always (1 week≥3 times)	14 (6.8%)	9 (4.2%)	
	almost never	11 (5.3%)	5 (2.4%)	
PRB scores (M ± SD)	occasionally (0–1 times per week) sometimes (1–2 times per week)	10.64 ± 2.487	10.12 ± 2.589	0.038 *
LPRB (N, %)	often (3–4 times per week)	16 (3.8%)		0.793
	always (4–5 times per week)	23 (5.5%)		
	Severe anxiety	6 (1.4%)		
MPRB (N, %)	Mild anxiety	138 (33.0%)		
	Moderate anxiety	131 (31.3%)		
	Severe anxiety	35 (8.4%)		
HPRB (N, %)	Mild anxiety	31 (7.4%)		
	Moderate anxiety	31 (7.4%)		
	Severe anxiety	7 (1.7%)		

Significance at *p* < 0.05 is marked with one asterisk, and significance at *p* < 0.01 is marked with two asterisks. EPs indicate endometrial polyps, whereas NEPs represent nonendometrial polyps. BMI stands for body mass index. PRB indicate potentially risky beverages, LPRB indicate Low potentially risky beverages, MPRB indicate medium potentially risky beverages, HPRB indicate high potentially risky beverages. Continuous variables were analyzed using logistic regression to examine their association with the outcome variables, while categorical variables were evaluated using the chi-square test to assess differences in proportions between groups.

### The relationship between anxiety and EPs

Table 2 analyzes the relationship between different levels of anxiety and Eps. It can be concluded from the table that there is no statistical significance between the average cumulative score of anxiety level and Eps (*p* = 0.749), and there is no statistical significance between different levels of anxiety and Eps (*p* = 0.353). Table 1 also analyzes the relationship between LPRB, MPRB, HPRB and different anxiety levels, and the statistical result (*p* = 0.793) shows that there is no statistical significance between PRB intake and different anxiety levels. Based on the above results, we can conclude that anxiety is a universal phenomenon in women of childbearing age, and more than half of the people who participated in this study have some degree of anxiety. Appeal to the social audience of women’s mental health.

**Table 2:** Association between anxiety levels and the occurrence of endometrial polyps.

Group	Average	*p* value	None	Mild	Moderate	Severe	Total	*p* value
	Mean ± SD		N, %	N, %	N, %	N, %	N, %	
NEPs	5.388 ± 4.116	0.749	100	85	17	10	212	0.353
			47.20%	40.10%	8.00%	4.70%	100.00%	
EPs	5.255 ± 4.413		85	100	12	9	206	
			41.30%	48.50%	5.80%	4.40%	100.00%	

EPs indicate endometrial polyps, whereas NEPs represent nonendometrial polyps. Categorical variables were evaluated using the chi-square test to assess differences in proportions between groups.

Relationship Between PRB and EPs

The Spearman correlation test was employed to assess the relationship between five types of PRB and the incidence of EPs (Table 3). The analysis revealed that sugar-sweetened beverages (*p* = 0.020) and milk tea (*p* = 0.047) were significantly associated with the occurrence of EPs. These findings suggest that higher consumption of milk tea and sugar-sweetened beverages may increase the risk of developing EPs.

**Table 3:** Analysis of the correlation between five types of potentially risky beverages and the incidence of endometrial polyps.

Items		Endometrial polyps	Sugar-sweetened beverages	Carbonate beverages	Soybean milk	Coffee	Milk tea
Endometrial polyp	Spearman correlation	1					
	*p value*	/					
Sugar-sweetened beverages	Spearman correlation	0.153	1				
	*p value*	0.020*	/				
Carbonate beverages	Spearman correlation	0.076	0.380	1			
	*p value*	0.121	0.000**	/			
Soybean milk	Spearman correlation	−0.017	0.025	0.056	1		
	*p value*	0.726	0.613	0.251	/		
Coffee	Spearman correlation	0.018	0.194	0.146	0.047	1	
	*p value*	0.713	0.000**	0.003**	0.348	/	
Milk tea	Spearman correlation	0.097	0.575	0.397	0.071	0.232	1
	*p value*	0.047*	0.000**	0.000**	0.146	0.000**	/

Significance at *p* < 0.05 is marked with one asterisk, and significance at *p* < 0.01 is marked with two asterisks. The Spearman correlation test was used to analyze the relationships among Eps and different kinds of drinks.

This study employs logistic regression to investigate the relationship between PRB intake and the risk of developing EPs (Table 4). In the analysis of unadjusted covariates, the results indicate that the MPRB drinks has an odds ratio (OR) of 2.026 (95% CI: 1.048–3.918, *p* = 0.036), whereas the high-income group has an OR of 2.452 (95% CI: 1.123–5.351, *p* = 0.024). Both groups had odds ratios greater than 1, suggesting that PRB intake may be associated with an elevated risk of ectopic pregnancies (EPs). Model 1 adjusts for age, body mass index, marital status, and other relevant factors, and the results remain consistent with the unadjusted analysis. Model 2 adjusts for age, body mass index, marital status, education level, household income, sexual history, pregnancy history, history of miscarriage and childbirth, and smoking and drinking habits. The results remain consistent with those of the previous models. Overall, the findings suggest that MPRB and HPRB intake are associated with an elevated risk of EPs, with odds ratios of 2.348 (95% CI: 1.153–4.780, *p* = 0.019) and 2.905 (95% CI: 1.2466.769, *p* = 0.0014), respectively.

**Table 4:** Regression analysis of logistics between potentially risky beverages and endometrial polyps.

Model	Beverage intake								
	Low			Moderate			High		
	OR	95% CI	*p value*	OR	95% CI	*p value*	OR	95% CI	*p value*
Crude	Ref.			2.026	1.048–3.918	0.036 *	2.452	1.123–5.351	0.024 *
Model 1				2.132	1.066–4.262	0.032 *	2.841	1.247–6.475	0.013 *
Model 2				2.348	1.153–4.78	0.019 *	2.905	1.246–6.769	0.014 *

Crude: Unadjusted. Model 1: Adjusted age, BMI, Marital status. Model 2: Adjusted age, BMI, marital status, Degree of education, Per capita monthly household income, sexual life, whether pregnant or not, history of abortion, childbearing history, smoke and drinking history. Significance at *p* < 0.05 is marked with one asterisk. Potentially risky beverages with a cumulative rating of 5–8 are considered “Low,” those with a cumulative rating of 9–12 are considered “Moderate,” and those with a cumulative rating of 13–25 are considered “High.” The binary logistic regression is used to regression the bicategorical variables, and the corresponding covariables are adjusted.

## Discussion

This case–control study found that high frequency consumption of PRB was associated with an increased prevalence of EPs. This is the first paper on the relationship between potentially risky beverage intake and the prevalence of EPs. We also explored the association between different levels of anxiety and EPs, which has certain guiding implications for women’s health.

As shown in Table 2 and Figure 2, despite the lack of statistical significance between anxiety levels and EPs (*p* = 0.353), over half of the women in both the EPs group (58.7%) and the NEPs group (52.8%) exhibited varying degrees of anxiety. Anxiety is very common among women and affects their mental health (21). Chronic anxiety can increase the risk of more serious diseases, such as major depressive episodes (22), cardiovascular disease (23), reproductive and postpartum health (24), and menopausal health (25). Dora Koller et al. reported a correlation between endometriosis and women’s mental health (26), and Rooney KL studies revealed a correlation between anxiety and infertility in women (27). Although our study did not identify a significant association between anxiety levels and EPs, the fact that more than half of the participants experienced anxiety highlights the need for increased societal focus on the mental health of women.

We conducted a comprehensive assessment of beverage intake on the basis of beverage type and frequency. The PRB included in this assessment included carbonated drinks, soy milk, sugary beverages, milk tea, and coffee. Current research indicates that excessive consumption of sugar-sweetened beverages is associated with the onset of various diseases. The World Health Organization (WHO) reported that high sugar intake increases the risk of dental caries, overweight and obesity. Additional studies have shown that the consumption of sugar-sweetened beverages can increase the risk of chronic diseases, including diabetes, cardiovascular disease, gout, cancer, and premature death, thereby increasing the overall burden of disease (28). A recent review of the evidence regarding the health outcomes of sugar intake, which comprehensively summarized the relationship between dietary sugar intake and multisystem diseases, indicated that high dietary sugar intake typically has a detrimental effect on health, particularly in its association with metabolic diseases (29). The highest level of evidence was for effects on body weight, followed by gout, HDL cholesterol, and metabolic syndrome (12). Currently, the relationship between excessive sugar intake and EPs remains inadequately understood; however, metabolic disturbances associated with high sugar consumption may contribute to an increased risk of EPs. Excessive sugar intake, for example, can lead to obesity, and adipose tissue in obese women secretes elevated levels of estrogen. This excess estrogen can stimulate endometrial hyperplasia, thereby increasing the risk of polyp formation (30). Furthermore, excessive sugar intake may contribute to the development of insulin resistance, which is closely associated with endocrine disorders, including polycystic ovary syndrome (31). A high-sugar diet promotes the occurrence of chronic low-grade inflammation in the body; especially under hyperglycemic conditions, the body’s immune system may be disrupted, and more proinflammatory cytokines may be produced (32). Long-term inflammation may lead to abnormal hyperplasia of the endometrium and the formation of polyps (33).

Milk tea has become a highly popular daily beverage in China. Unlike traditional tea, milk tea contains not only tea but also relatively high levels of sugar, creamers, saturated fat, and additives. High consumption of sugar and creamers increases the risk of obesity, whereas excessive saturated fat negatively impacts lipid metabolism and increases cardiovascular risk (34). The correlation analysis revealed that milk tea intake was significantly associated with EPs (OR = 0.097, *p* = 0.047), which aligns with the adverse health effects associated with sugar-sweetened beverages.

Our study revealed no significant associations between coffee, soy milk, or carbonated beverages and the prevalence of EPs. However, this did not imply that these beverages are either safe or harmful. Current research on coffee presents mixed findings. Caffeine is the primary component of coffee. One study examining the relationship between caffeine and estrogen levels reported that increased caffeine intake may increase estrogen levels (14). Caffeine consumption is associated with favorable effects on a variety of health outcomes in women, including some diseases related to estrogen metabolism (35). Moreover, caffeine intake is often linked to increased social stress, greater life burdens, reduced physical activity, and poor sleep quality. Unhealthy lifestyles, characterized by poor psychological well-being, inadequate sleep, and a lack of exercise, are frequently associated with a higher incidence of chronic diseases (36). Further cohort studies and gene-related research are necessary to investigate the potential causal relationship between coffee consumption and endometrial health. This study revealed that soy milk and other soy products contain phytoestrogens, primarily isoflavones, which have a weak effect, and typical dietary intake does not result in significant endocrine disruption (37). Conversely, these phytoestrogens may have a beneficial effect on regulating estrogen levels in the body, acting as “antiestrogens” when estrogen levels are excessively high. Alternatively, when estrogen levels are low, these compounds may exert a mild estrogenic effect, aiding in the regulation of hormonal balance (38). Additionally, our correlation analysis indicates that there may be a beneficial relationship between the two (OR = −0.17, *p* = 0.726).

Sugar-sweetened carbonated beverages, commonly referred to as soft drinks, are made by carbonating liquid with carbon dioxide gas and sweetening the mixture. Their main components include white sugars, coloring agents, flavorings, carbonated water, acidulants, and sweeteners (39). Carbonated beverages are widely consumed in daily life. An increasing body of research has identified a strong link between the long-term consumption of carbonated beverages and numerous health issues (40), including excessive caloric intake, reduced consumption of dairy products and other nutrients, and a heightened risk of dental caries, osteoporosis, depression, behavioral disorders, and other psychological problems (41). This consumption is also closely associated with obesity, diabetes, metabolic syndrome, gout, and high blood pressure, particularly in children and adolescents (42), gout is closely associated with hypertension (43). A case–control study examining the relationship between carbonated beverages and obesity revealed a monotonic, linear dose–response association between Sugar-sweetened carbonated beverages consumption and obesity (*p* = 0.02), as well as increases in BMI and percentage of body fat mass (39). Sugar-sweetened beverage consumption and age at menarche in a prospective study of American girls (44). Frequent consumption of carbonated beverages is associated with earlier menarche, and a 1-year decrease in the age of menarche correlates with a 5% increased risk of breast cancer (45, 46). Suggesting a correlation between carbonated beverage consumption and women’s health. Carbonated beverage consumption may induce a state of chronic inflammation in the body, which can result in the production of additional proinflammatory cytokines (47, 48). A chronic inflammatory environment can contribute to the development of EPs (49).

### Limitations and prospects of the current study

One of the strengths of this case–control study is that it is the first to examine the relationship between the incidence of EPs and the frequency of consumption of PRB in women of childbearing age, while also exploring the connection between EPs and different levels of anxiety. In everyday life, understanding the intake of PRB among women of reproductive age holds important guiding significance, emphasizing the need for society to pay attention to the dietary habits and mental health of this population.

However, the study has several limitations. The participants were patients from the gynecological outpatient department at Chengdu University of Traditional Chinese Medicine, and the cases were sourced from a specific region, which means the findings primarily reflect the relationship between PRB intake and EPs among women in southwest China. Despite this, all patients with EPs met international diagnostic criteria, and strict inclusion criteria were applied to ensure the accuracy of the results.

The questionnaire was designed to assess the frequency of PRB consumption but did not include a quantitative evaluation of total beverage intake, which limited the ability to analyze the linear correlation between beverage consumption and EPs. However, by categorizing beverage intake based on frequency, it was divided into low, medium, and high levels, which better represented the actual intake patterns to some extent, providing a crucial analytical foundation for investigating the relationship between beverage intake levels and the occurrence of EPs.

Furthermore, since age and reproductive history may influence the risk of EPs, this study controlled for these potential confounders by using logistic regression to ensure the accuracy of the findings. To mitigate recall bias, a contradictory option was included in the questionnaire to verify the accuracy of responses. If contradictory answers were identified, the corresponding data were excluded to ensure the integrity of the dataset.

Despite these limitations, the study was designed in strict accordance with the STROBE observational study guidelines, and the statistical analysis was properly conducted.

## Conclusion

Based on the results, there was a significant correlation between increased intake of PRB and a higher prevalence of EPs. Additionally, more than half of the participants exhibited varying degrees of anxiety, underscoring the importance of addressing women’s health concerns in society. This study also provides a valuable reference for future research aiming to quantitatively evaluate the linear relationship between beverage consumption and EPs.

## Data Availability

The datasets presented in this study can be found in online repositories. The names of the repository/repositories and accession number(s) can be found in the article/Supplementary material.

## Footnotes

^1^ https://app.diagrams.net/
